# Toxic Characteristics and Action Mode of the Mixotrophic Dinoflagellate *Akashiwo sanguinea* on Co-Occurring Phytoplankton and Zooplankton

**DOI:** 10.3390/ijerph19010404

**Published:** 2021-12-30

**Authors:** Xiaoer Wu, Ying Yang, Yeyin Yang, Ping Zhong, Ning Xu

**Affiliations:** 1 Institute of Hydrobiology, Jinan University, Guangzhou 510632, China; wuxiaoer@zjblab.com (X.W.); yangying@stu2019.jnu.edu.cn (Y.Y.); yeyin.x.yang@gsk.com (Y.Y.); 2 Key Laboratory of Eutrophication and Red Tide Prevention of Guangdong Higher Education Institutes, Jinan University, Guangzhou 510632, China; 3 Marine Resources Big Data Center of South China Sea, Southern Marine Science and Engineering Guangdong Laboratory, Zhanjiang 524013, China

**Keywords:** harmful algal blooms, dinoflagellate, phytoplankton, zooplankton, mixotrophy, cell contact

## Abstract

The mixotrophic dinoflagellate *Akashiwo sanguinea* frequently forms harmful algal blooms around the world and has caused massive deaths of shellfish, finfish and birds, yet its toxic mechanism is still unclear. In this study, toxic effects of *A. sanguinea* on co-culturing phytoplankton and zooplankton were investigated. The results showed that sonicated cultures of *A. sanguinea* JX13 and JX14, isolated from the Pearl River Estuary, had a significant lethal effect on the rotifer *Brachionus plicatilis*, with the highest mortality rate of 80%. The highest inhibition rates of *A. sanguinea* cultures JX13 (90%) and JX14 (80%) on *R. salina* were much higher than that of AS2 (20%). Toxicity varied with the growth stage, during which *A. sanguinea* cells in the exponential stage showed the highest toxicity (40%), while *A. sanguinea* filtrate had the highest toxicity (10%) in the decline stage. The action mode of *A. sanguinea* toxicity on plankton was explored through an osmotic membrane culture device. It was found that *A. sanguinea* JX13 displayed an inhibitory effect on coexisting phytoplankton, whether they had contact or not, but the inhibition rate increased by 25% with contact. A lethal effect of *A. sanguinea* JX13 on rotifer *Brachionus plicatilis* was observed only in contact treatment. This study suggests that direct contact is the key action mode to trigger the release of toxins and induce toxic effects of *A. sanguinea* on co-occurring plankton.

## 1. Introduction

*Akashiwo sanguinea* is a mixotrophic dinoflagellate that adapts to wide ranges of temperature (10–30 °C) and salinity (5–40) [1], utilizes various forms of N sources, including urea, ammonia and nitrate [2], and forms cysts under unfavorable conditions [3]. In recent decades, this species has formed harmful algal blooms (HABs) in coastal waters worldwide, including in Australia [4], North America [5], Europe [6], South America [7] and Asia [8,9], resulting in a large number of deaths of fish, shellfish and birds [7,10]. In August 2009, a large-scale *A. sanguinea* bloom broke out in the northeast Pacific off the coast of Washington and Oregon, which lasted for several months, causing the death of thousands of seabirds [11]. In China, the first record of *A. sanguinea* bloom happened on the coast of Shandong Province in 1998, resulting in the death of a large number of cultured scallops [8]. Since then, *A. sanguinea* has broken out recurrently along the coast of Fujian Province, Zhejiang Province and Guangdong Province, becoming the most common HAB species in China [12]. Thus far, a total of more than 30 recorded *A. sanguinea* blooms have occurred in four major sea areas of China, with a total affected area of 2098 km^2^, causing huge economic losses and ecological hazards [12].

Although *A. sanguinea* has caused blooms all over the world, resulting in mass mortality of marine organisms [9,10], this species has still been considered a non-toxic dinoflagellate for a long time. Horner et al. suggested *A. sanguinea* causes mortality of oysters and spot prawns due to mechanical damage or oxygen stress [5]. Another study showed that *A. sanguinea* is able to secrete mucus to cover the gills of fish and suffocate them [13]. However, few studies found that *A. sanguinea* is highly toxic to larvae of pacific oysters and Japanese littleneck clams [14]. Recently, laboratory studies showed that *A. sanguinea* has a significant lethal effect on shrimp, bivalve and finfish [15] and can inhibit the growth of coexisting phytoplankton (*Phaeocystis globosa*, *Scrippsiella trochoidea* and *Rhodomonas salina*) [16]. Another research found that *A. sanguinea* has strong hemolytic toxicity, with a hemolytic activity of 9.76 × 10^−5^ HU/cell [17]. Therefore, this species is potentially toxic to marine organisms in different trophic levels, yet the mechanism and action mode of *A. sanguinea* toxicity are still unknown.

This study investigated the effects and variations of the toxicity of *A. sanguinea* on co-culturing phytoplankton and zooplankton and further explored the action mode of *A. sanguinea* toxicity on target organisms through an osmotic membrane culture device. The results will be meaningful for understanding the competitive strategy of *A. sanguinea*.

## 2. Materials and Methods

### 2.1. Algal Cultures and Conditions

*A. sanguinea* JX13 and JX14, with GenBank serial numbers of KF793277 and KF793278, respectively, were isolated from field samples from Daya Bay, Guangdong Province, China and identified by morphology (light microscope) and LSU rDNA sequence [15]. *A. sanguinea* AS2 was isolated from Chesapeake Bay (Norfolk, VA, USA) [18]. *Rhodomonas salina* CCMP1319 was provided by Dr. Christopher J. Gobler of Stony Brook University. *Prorocentrum micans* JX8 was isolated from the South China Sea.

All algae strains were cultured in a light incubator (GXZ intelligent light incubator, Ningbo, China) and grown in sterile, silicate-free f/2 medium [19], which was prepared with filtered f/2 medium stock (passing through 0.22 micron filters) and autoclaved seawater with a salinity of 28 ± 1 (unless otherwise noted). Cultures were maintained at constant temperature (19 ± 1 °C) and irradiance (100 μmol m^−2^ s^−1^) in a 12 h light/12 h dark cycle.

The rotifer *Brachionus plicatilis* (Futian Biotechnology Co., Ltd., Ningbo, China, 4 days old) was cultured with autoclaved seawater at 23 ± 1 °C at a salinity of 28 ± 1 in a 12 h light/12 h dark cycle and fed with *Chlorella pyrenoidosa* (Futian Biotechnology Co., Ltd., Ningbo, China). Rotifers of similar size were filtered into autoclaved seawater before the experiment and not fed during the experiment.

**Figure 1:**
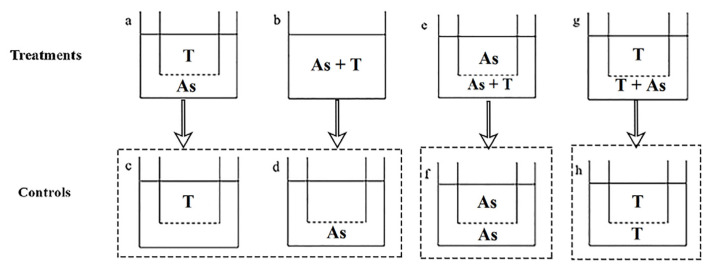
Experimental design for action mode of *Akashiwo sanguinea* toxicity on phytoplankton and zooplankton. Treatments: (**a**,**b**,**e**,**g**). Controls: (**c**,**d**,**f**,**h**). (**a**): non-contact; (**b**): contact; (**e**,**g**): half-contact. (**c**,**d**) were the controls of (**a**,**b**); (**f**) was the control of (**e**); (**h**) was the control of (**g**). As: *A. sanguinea*; T: *Rhodomonas salina* (Section 2.4.1) and *Brachionus plicatilis* (Section 2.4.2), respectively. The dotted line below the insert of the six-well plate means 3 μm pore size membrane.

Co-cultures were carried out using an osmotic membrane device. The 3 μm pore diameter polycarbonate membrane insert (Corning Inc., New York, NY, USA) was suspended on each well of the six-well plate, with the bottom of insert located in the middle layer of the medium (shown in Figure 1).

### 2.2. Study 1: Toxic Effects of A. sanguinea on Co-Occurring Zooplankton

Mid-exponential phase (4 days after inoculation) *A. sanguinea* JX13, JX14 and AS2 cultures (500 mL) were sonicated at 100% power output, 5 s ON/5 s OFF pulses for 20 min in an ice bath and examined under microscope to ensure all cells were broken. The sonicated cultures were diluted using f/2-Si medium to three cell densities equivalent to 5000, 8000 and 11,000 cells mL^−1^. The bioassay was set up in a six-well plate (Corning, NY, USA) with a final volume of 8 mL per well. *B. plicatilis* (initial density: 10 individuals well^−1^) was added to sonicated cultures of *A. sanguinea* and maintained for 36 h. A total of 10 individuals of *B. plicatilis* in sonicated *P. micans* JX8 cultures (three corresponding concentrations mentioned above) and f/2-Si medium were used as controls. Each treatment and control was prepared in triplicate (*n* = 3). Every 6 h, the number of surviving rotifers was recorded, and dead ones were removed.

### 2.3. Study 2: Toxic Effects of A. sanguinea on Co-Occurring Phytoplankton

#### 2.3.1. Variations of Toxicity among *A. sanguinea* Strains

Mid-exponential (4 days after inoculation) *A. sanguinea* JX13, JX14 and AS2 (initial cell density: 1000, 3000 and 5000 cells mL^−1^, respectively) were co-cultured with *R. salina* CCMP1319 (initial cell density: 1000 cells mL^−1^) for 72 h. The experiment was conducted in a six-well plate with a total volume of 10 mL per well. Monocultures of *A. sanguinea* and *R. salina* were used as controls, while treatments and controls were established in triplicate (*n* = 3). F/2-Si medium was added at the beginning of the experiment to ensure sufficient nutrition during the experiment. At 24, 48 and 72 h, 1 mL of samples was fixed in 2% Lugol’s solution, and cell densities of *A. sanguinea* and *R. salina* were calculated using light microscopy (CX41, Olympus Corporation, Tokyo, Japan). pH was measured (PHB-3, Shanghai Sanxin Instrument Factory, Shanghai, China) at the beginning and end of the experiment.

#### 2.3.2. Variations of *A. sanguinea* Toxicity among Different Growth Stages and between Extra- and Intra-Cellular Fractions

Exponential, stationary and decline-phase (6, 9 and 12 days after inoculation, respectively) *A. sanguinea* JX14 cultures at a salinity of 22 ± 1 were centrifuged at 1500× *g*, 4 °C for 10 min to separate cell-free supernatant and cell pellet. The supernatant was collected, and the cell pellet was washed with autoclaved seawater and sonicated at 100% power output, 5 s ON/5 s OFF pulses for 15 min in an ice bath. The experiment was conducted in a six-well plate with a total volume of 10 mL per well. The supernatant and sonicated cell pellet of *A. sanguinea* JX14 were added to *R. salina* CCMP1319 culture, and monoculture of *R. salina* was used as control. Concentrations of supernatant and sonicated cell pellet of *A. sanguinea* JX14 were equivalent to a cell density of 4000 cells mL^−1^ and the concentration of *R. salina* CCMP1319 was 1500 cells mL^−1^. Treatment and control were established in triplicate (*n* = 3). F/2-Si medium was added at the beginning of the experiment to ensure sufficient nutrition during the experiment. At 24 and 48 h, 1 mL of samples and controls was fixed in 2% Lugol’s solution, and cell density of *R. salina* was calculated with light microscopy (CX41, Olympus Corporation, Tokyo, Japan).

### 2.4. Study 3: Action Mode of A. sanguinea Toxicity on Phytoplankton and Zooplankton

#### 2.4.1. Action Mode of *A. sanguinea* Toxicity on Phytoplankton

Mid-exponential (4 days after inoculation) *A. sanguinea* JX13 and AS2 were co-cultured with *R. salina* CCMP1319 for 48 h in three different contact types (contact, non-contact and half-contact), and monocultures of JX13, AS2 and CCMP1319 were used as controls. The experiment was conducted in a six-well plate and appended with polycarbonate membrane inserts (3 μm pore diameter, Corning, NY, USA), with a total volume of 9 mL for each well. The specific experimental design is shown in Figure 1. Initial cell densities of *A. sanguinea* and *R. salina* were 3000 cells mL^−1^ and 1000 cells mL^−1^, respectively. Each treatment and control was established in triplicate (*n* = 3), and f/2-Si medium was added at the beginning of the experiment to ensure sufficient nutrition during the experiment. At 24 and 48 h, 0.5 mL samples were fixed in 2% Lugol’s solution, and cell densities of *A. sanguinea* and *R. salina* were calculated with light microscopy (CX41, Olympus Corporation, Tokyo, Japan).

#### 2.4.2. Action Mode of *A. sanguinea* Toxicity on Zooplankton

Mid-exponential (4 days after inoculation) *A. sanguinea* JX13 and AS2 were co-cultured with *B. plicatilis* for 48 h in three different contact types (contact, non-contact and half-contact), and monocultures of JX13, AS2 and *B. plicatilis* in f/2-Si medium were used as controls. The experiment was conducted in a six-well plate appended with polycarbonate membrane insert (3 μm pore diameter, Corning, NY, USA), with a total volume of 8 mL for each well (experimental design is shown in Figure 1. The initial cell density of *A. sanguinea* was 8000 cells mL^−1^, and the initial number of *B. plicatilis* in each well was 8. Each treatment and control was established in triplicate (*n* = 3), and f/2-Si medium was added at the beginning of the experiment to ensure sufficient nutrition during the experiment. At 24 and 48 h, surviving rotifers were enumerated, dead ones were removed, and 0.5 mL samples were fixed in 2% Lugol’s solution. Cell density of *A. sanguinea* was calculated with light microscopy (CX41, Olympus Corporation, Tokyo, Japan).

### 2.5. Statistical Analysis

Data were analyzed using one-way ANOVA. Significance was defined at *p* < 0.05, and extreme significance was defined at *p* < 0.01. All statistical analyses were performed using SPSS software (version 19) (IBM, New York, NY, USA) and Origin software (version 9) (OriginLab Corporation, Northampton, MA, USA).

Inhibition rate = (1 − *N_treatment_*/*N_control_*) × 100% (1)

Relative cell density = *N_treatment_*/*N_control_* × 100% (2)

where *N_treatment_* and *N_control_* are numbers of target alga in treatment and control, respectively.

## 3. Results

### 3.1. Toxic Effects of A. sanguinea on Co-Occurring Zooplankton

**Figure 2:**
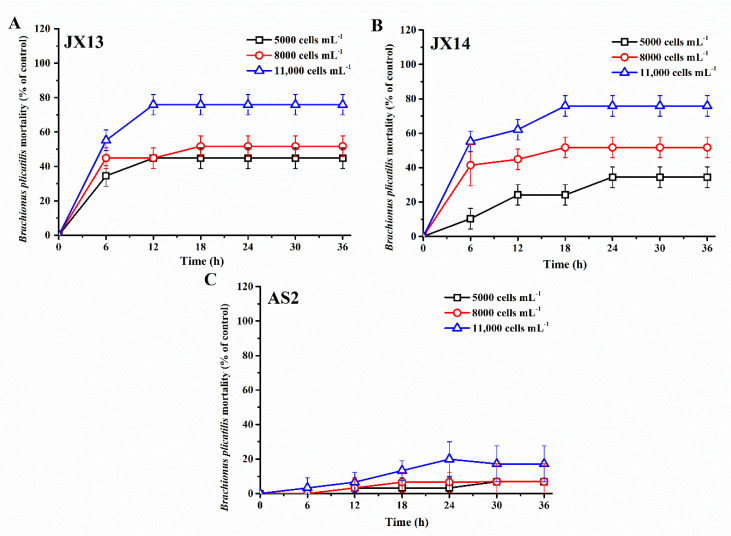
Mortality of *Brachionus plicatilis* exposed to sonicated cultures of *Akashiwo sanguinea*. (**A**) JX13, (**B**) JX14, (**C**) AS2. The initial number of *B. plicatilis* was 10 for each well. Results are expressed in triplicate ± standard deviation (SD).

Both *A. sanguinea* JX13 and JX14 showed significant lethal effects on the rotifer *B. plicatilis*, and rotifer mortality increased with cell density of *A. sanguinea* and duration of the experiment, reaching a plateau after 12–18 h (JX13) or 18-24 h (JX14) (*p* < 0.05, Figure 2A,B). Specifically, at the end of the experiment, rotifer mortalities in the highest cell-density treatments (11,000 cells mL^−1^) of *A. sanuinea* JX13 and JX14 were about 80%, which is significantly higher than that of the sonicated JX8 culture control (3%) and medium control (3%) (*p* < 0.01, Figure 2A,B). At the lowest *A. sanguinea* cell density (5000 cells mL^−1^), rotifer mortality in the JX13 treatment was significantly higher than that in the JX14 treatment (*p* < 0.05). In contrast with *A. sanguinea* JX13 and JX14, AS2 only caused slight mortality in rotifer, with the highest mortality of 20% in the highest cell-density treatment (11,000 cells mL^−1^) (*p* < 0.05, Figure 2C). There was no significant difference observed in rotifer mortality between sonicated JX8 culture control and medium control (*p* > 0.05).

### 3.2. Toxic Effects of A. sanguinea on Co-Occurring Phytoplankton

#### 3.2.1. Variations of Toxicity among *A. sanguinea* Strains

**Figure 3:**
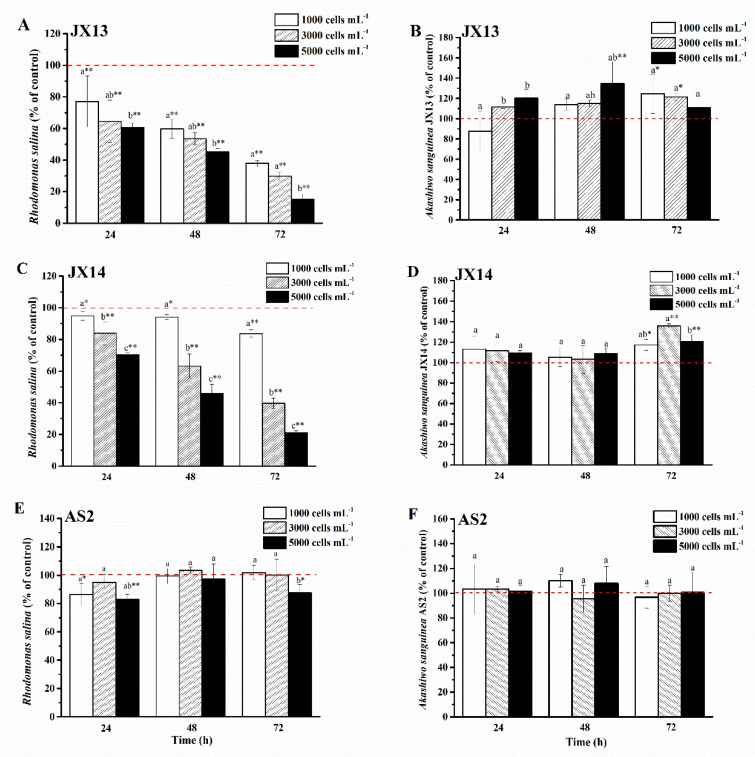
Strains of *Akashiwo sanguinea* JX13, JX14 and AS2 co-cultured with *Rhodomonas salina* CCMP1319. (**A**,**B**): JX13 co-cultured with *R. salina*; (**C**,**D**): JX14 co-cultured with *R. salina*; (**E**,**F**): AS2 co-cultured with *R. salina*. Initial cell density of *R. salina* was 1000 cells mL^−1^. Results are expressed in triplicate ± standard deviation (SD). Different lower-case letters (a, b, c) indicate significant differences (*p* < 0.05) between treatments at the same time; * and ** indicate significant differences (*p* < 0.05 and *p* < 0.01, respectively) between treatment and control.

Both *A. sanguinea* JX13 and JX14 showed significant growth inhibition in a dose-dependent manner on co-cultured *R. salina* CCMP1319 (Figure 3A,C), while *A. sanguinea* AS2 had a slight inhibitory effect (Figure 3E). At the highest *A. sanguinea* cell density (5000 cells mL^−1^), inhibition rates of JX13 (90%) and JX14 (80%) were significantly higher than that of AS2 (20%) at 72 h (*p* < 0.01). In addition, at the lowest *A. sanguinea* cell density (1000 cells mL^−1^), inhibition rates of JX13 (60%) were significantly higher than those of JX14 (20%) (*p* < 0.01), indicating that toxicity is higher in JX13 than in JX14.

It is worth noting that when co-cultured with *R. salina*, cell densities of *A. sanguinea* JX13 and JX14 increased significantly compared with the control, with a maximum increasing range of about 40% (*p* < 0.01, Figure 3B,D), while no significant change was observed in the relative cell density of AS2 (*p* > 0.05, Figure 3F).

During the experiment, pH values varied from 7.9 ± 0.3 to 8.3 ± 0.2.

#### 3.2.2. Variations of *A. sanguinea* Toxicity among Different Growth Stages and between Extra- and Intra-Cellular Fractions

**Figure 4:**
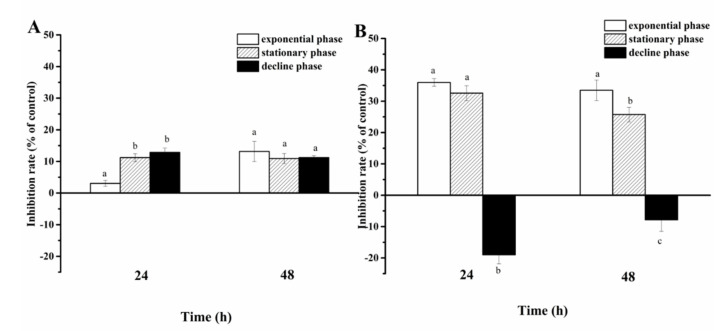
Inhibition rates of *Akashiwo sanguinea* JX14 at different growth phases on *Rhodomonas salina* CCMP1319. (**A**): cell-free filtrate of *A. sanguinea* JX14; (**B**): cell pellet of *A. sanguinea* JX14. Corresponding concentration and initial cell density of *A. sanguinea* JX14 and *R. salina* were 4000 cells mL^−1^ and 1500 cells mL^−1^, respectively. Results are expressed in triplicate ± standard deviation (SD). Different lower-case letters (a, b, c) indicate significant difference (*p* < 0.05) between treatments at the same time.

Cell-free filtrate of *A. sanguinea* JX14 showed significant inhibitory effects on *R. salina* CCMP1319 (*p* < 0.05), and inhibition rates of filtrate in stationary (11%) and decline (13%) phases were significantly higher than those in the exponential phase (3%) at 24 h (*p* < 0.05, Figure 4A).

The inhibition rate of JX14 cell pellets in exponential phase (33%) was significantly higher than that in stationary phase (26%) at 48 h (*p* < 0.05), while decline-phase cell pellets showed a growth-promoting effect on *R. salina*.

The growth-inhibition effects of JX14 cell pellet on *R. salina* in exponential and stationary phases were about 3 times those of associated filtrates.

### 3.3. Action Mode of A. sanguinea Toxicity on Phytoplankton and Zooplankton

#### 3.3.1. Action Mode of *A. sanguinea* Toxicity on Phytoplankton

(1) Contact and non-contact coculture

**Figure 5:**
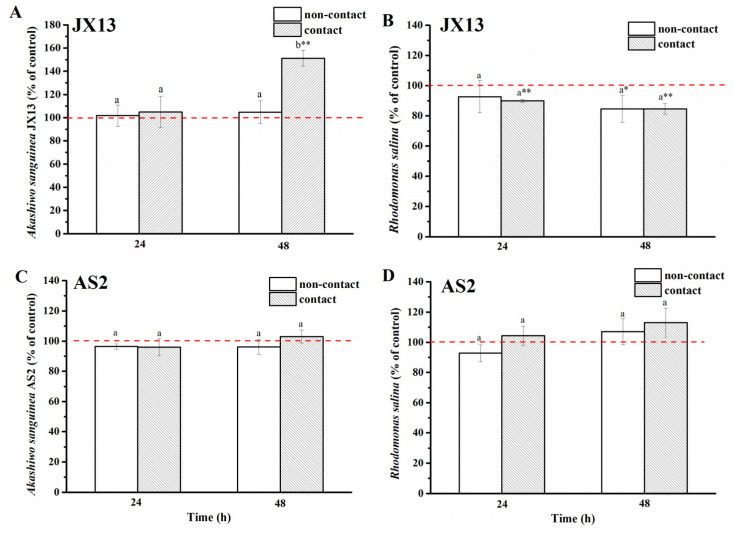
Contact and non-contact coculture of *Akashiwo sanguinea* (JX13, AS2) and *Rhodomonas salina* CCMP1319. (**A**,**B**): *A. sanguinea* JX13 co-cultured with *R. salina*; (**C**,**D**): *A. sanguinea* AS2 co-cultured with *R. salina*. Initial cell densities of *A. sanguinea* and *R. salina* were 3,000 cells mL^−1^ and 1,000 cells mL^−1^, respectively. Results are expressed in triplicate ± standard deviation (SD). Different lower-case letters (a, b) indicate significant differences (*p* < 0.05) between treatments at the same co-culture times; * and ** indicate significant differences (*p* < 0.05 and *p* < 0.01, respectively) between treatment and control.

In terms of toxic *A. sanguinea* strain JX13, its relative cell density increased by about 50%, while *R. salina* concentration decreased by about 20% at 48 h (*p* < 0.01, Figure 5A,B) in the contact treatment. In contrast, no significant change was observed in JX13 cell density, while *R. salina* showed a significant decrease (about 20%, *p* < 0.05) at 48 h in the non-contact treatment. There was a significant difference in the relative cell density of *A. sanguinea* JX13 between the two coculture methods (at 48 h, *p* < 0.05, Figure 5A), but no significant difference was found in the relative density of *R. salina* (Figure 5B).

As to the nontoxic (or low-toxic) strain, *A. sanguinea* AS2, there was no significant difference observed in the cell density of AS2 and *R. salina* compared with controls, regardless of contact or non-contact conditions, and no significant difference was found in the cell density of AS2 and *R. salina* between contact and non-contact treatments (*p* > 0.05, Figure 5C,D).

(2) Half-contact coculture

**Figure 6:**
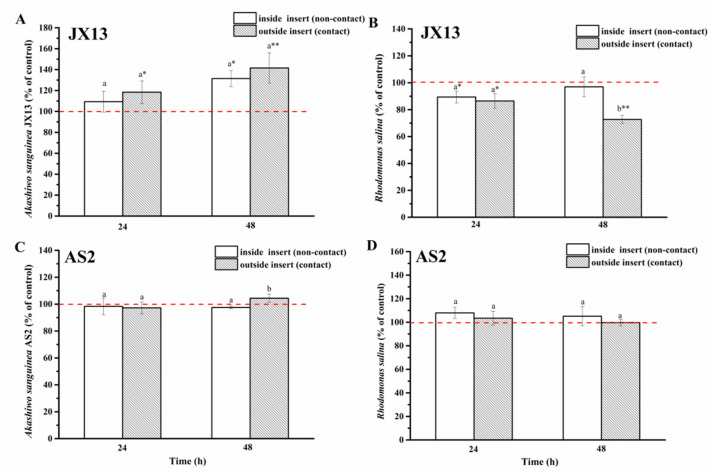
Half-contact coculture of *Akashiwo sanguinea* (JX13, AS2) and *Rhodomonas salina* CCMP1319. (**A**,**B**): *A. sanguinea* JX13 co-cultured with *R. salina*; (**C**,**D**): *A. sanguinea* AS2 co-cultured with *R. salina*. Initial cell densities of *A. sanguinea* and *R. salina* were 3000 cells mL^−1^ and 1000 cells mL^−1^, respectively. Results are expressed in triplicate ± standard deviation (SD). Different lower-case letters (a, b) indicate significant differences (*p* < 0.05) between treatments at the same co-culture times; * and ** indicate significant differences (*p* < 0.05 and *p* < 0.01, respectively) between treatment and control.

When contact and non-contact coexisted (half-contact coculture), relative cell densities of *A. sanguinea* JX13 inside (non-contact) and outside (contact) of the insert increased significantly, by about 30% (*p* < 0.05) and 40% (*p* < 0.01), respectively, at 48 h, with no significant difference between them (Figure 6A). On the other hand, relative cell density of *R. salina* inside the insert (non-contact) decreased slightly at 24 h (*p* < 0.05), while the relative cell density of *R. salina* outside the insert (contact) decreased continuously with longer coculture time (decreased by 30% at 48 h, *p* < 0.01) (Figure 6B). There was a significant difference in *R. salina* cell density inside and outside of the insert at 48 h (*p* < 0.05).

In the AS2 treatment, there was no significant difference in cell density of AS2 and *R. salina* compared with controls (*p* > 0.05, Figure 6C,D).

#### 3.3.2. Action Mode of *A. sanguinea* Toxicity on Zooplankton

(1) Contact and non-contact coculture

**Figure 7:**
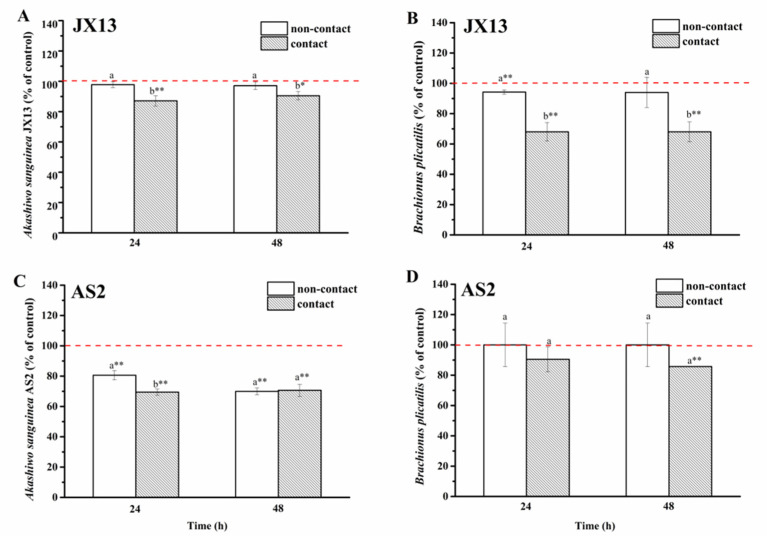
Contact and non-contact coculture of *Akashiwo sanguinea* (JX13, AS2) and *Brachionus plicatilis*. (**A**,**B**): *A. sanguinea* JX13 co-cultured with *B. plicatilis*; (**C**,**D**): *A. sanguinea* AS2 co-cultured with *B. plicatilis*. Initial cell density of *A. sanguinea* was 8000 cells mL^−1^, and the initial number of *B. plicatilis* was 8 for each well. Results are expressed in triplicate ± standard deviation (SD). Different lower-case letters (a, b) indicate significant differences (*p* < 0.05) between treatments at the same co-culture times; * and ** indicate significant differences (*p* < 0.05 and *p* < 0.01, respectively) between treatment and control.

In the non-contact coculture treatment of *A. sanguinea* JX13 and *B. plicatilis*, there was no significant difference observed in relative concentrations of both species at 48 h (*p* > 0.05, Figure 7A,B). In the contact coculture treatment, relative concentrations of JX13 and rotifer decreased by about 10% (*p* < 0.05) and 30% (*p* < 0.01), respectively. There were significant differences in relative concentrations of JX13 and *B. plicatilis* between contact and non-contact treatments (*p* < 0.05).

As for *A. sanguinea* AS2, the relative cell density decreased by 30% after non-contact co-cultured with *B. plicatilis* for 48 h (*p* < 0.01), while no significant change was observed in the relative density of *B. plicatilis* (Figure 7C,D). In the contact treatment, the relative concentrations of AS2 and *B. plicatilis* decreased by about 30% and 14% (*p* < 0.01, Figure 7C,D), respectively. At the end of the experiment, there was no significant difference observed in relative densities of AS2 and rotifer between the non-contact and contact treatments (at 48 h, Figure 7C,D).

(2) Half-contact coculture

**Figure 8:**
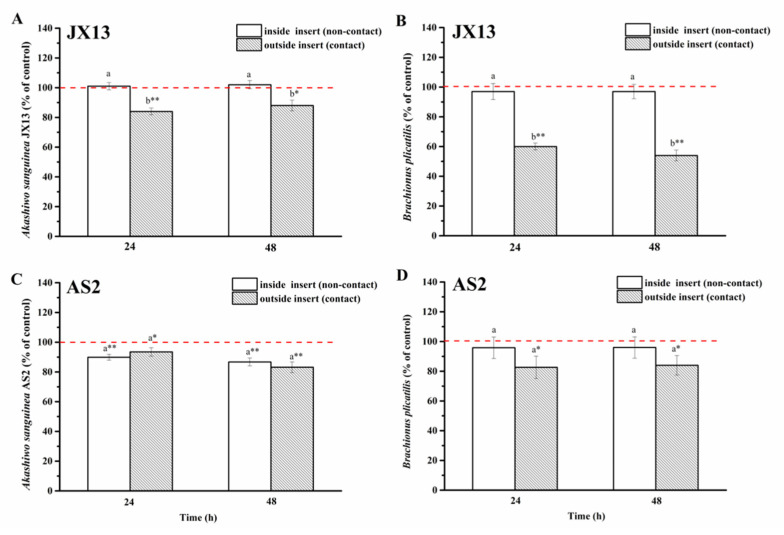
Half-contact coculture of *Akashiwo sanguinea* (JX13, AS2) and *Brachionus plicatilis*. (**A**,**B**): *A. sanguinea* JX13 co-cultured with *B. plicatilis*; (**C**,**D**): *A. sanguinea* AS2 co-cultured with *B. plicatilis*. Initial cell density of *A. sanguinea* was 8000 cells mL^−1^, and the initial number of *B. plicatilis* was 8 for each well. Results are expressed in triplicate ± standard deviation (SD). Different lower-case letters (a, b) indicate significant differences (*p* < 0.05) between treatments at the same co-culture times; * and ** indicate significant differences (*p* < 0.05 and *p* < 0.01, respectively) between treatment and control.

When co-cultured with *A. sanguinea* JX13, the relative density of *B. plicatilis* outside of the insert (contact) decreased by 50% compared with the control (*p* < 0.01, Figure 8B) at 48 h, while its relative density inside of the insert (non-contact) showed no significant change. On the other hand, the relative cell density of *A. sanguinea* JX13 outside the insert (contact) decreased by about 20% compared with the control (*p* < 0.05, Figure 8A), while no significant difference was observed in JX13 concentration inside of the insert (non-contact). There were significant differences in the relative density of *A. sanguinea* JX13 and *B. plicatilis* inside and outside of the insert (*p* < 0.05).

As for *A. sanguinea* AS2, its relative cell density decreased significantly whether inside (10%) or outside (20%) of the insert (*p* < 0.01, Figure 8C). At the end of the experiment, the relative density of *B. plicatilis* outside of the insert decreased by about 20%, while no change was observed in rotifer density inside of the insert (Figure 8D). There was no significant difference in the relative density of AS2 and *B. plicatilis* between the contact and non-contact groups (Figure 8D).

## 4. Discussion

### 4.1. Toxic Effects of A. sanguinea on Co-Occurring Plankton

Field studies have shown that *A. sanguinea* blooms have been responsible for the massive death or disease of fish, shellfish, seabirds and other marine organisms [7,10,20]. To this point, there was little understanding of the potential effects of this cosmopolitan HAB species on lower trophic levels, such as phytoplankton and zooplankton.

Study 1 investigated the toxicity of two Chinese strains of *A. sanguinea* (JX13 and JX14) and an American strain of *A. sanguinea* (AS2) to co-occurring zooplankton. Our results showed that sonicated cultures of *A. sanguinea* JX13 and JX14 exhibited acute toxicity to zooplankton *B. plicatilis*, with an IR_max_ of 80%, while the IR_max_ of *A. sanguinea* AS2 (20%) was significantly lower than that of *A. sanguinea* JX13 and JX14 (*p* < 0.01, Figure 2). Analogously, Yang et al. (2021) found that *A. sanguinea* had significant inhibiting effects on *R. salina*, *S. trochoidea* and *P. globosa* using a whole-cell coculture experiment [16]. Xu et al. (2017) found that sonicated *A. sanguinea* culture had lethal effects on brine shrimp *Artemia salina* through cell lysates of culture [15]. In addition, it was found that *A. sanguinea* bloom could change the zooplankton community structure significantly [21]. Therefore, these studies support that *A. sanguinea* species is able to produce toxins and has significant toxic effects on phytoplankton and zooplankton.

### 4.2. Variation Characteristics of A. sanguinea Toxicity

Study 2 investigated variations of toxicity among different *A. sanguinea* strains. The highest inhibition rates of *A. sanguinea* JX13 and JX14 (90%, 80%) on *R. salina* were significantly higher than those of *A. sanguinea* AS2 (20%) (*p* < 0.01). In addition, study 1 demonstrated that there are significant differences between the toxicity of sonicated *A. sanguinea* cultures JX13 and JX14 on *B. plicatilis* (Figure 2). Study 3 also provided evidence that the toxicity of *A. sanguinea* JX13 on *R. salina* and *B. plicatilis* was significantly higher than that of AS2 (Figure 5, Figure 6, Figure 7 and Figure 8). Our previous studies showed that there is a significant difference between the hemolytic activity of *A. sanguinea* JX14 and AS2 on rabbit erythrocytes (9.85 × 10^−5^ and 3.72 × 10^−5^ HU cell^−1^, respectively, *p* < 0.05) [15]. Variations in toxicity among *A. sanguinea* strains under the same cultural conditions suggest that genetic differences should be the fundamental factor regulating the production of *A. sanguinea* toxins.

We compared the toxicity of cell pellet and cell-free filtrate of *A. sanguinea* JX14 at different growth phases to that of *R. salina*. The results showed that the inhibition rates of cell pellet (40% at exponential and stationary phases) were much higher than those of cell-free filtrate (10%), which clearly demonstrates that the cells of *A. sanguinea* should be the source of toxins. Based on the inhibition rate of cell pellet on *R. salina*, it can be concluded that the exponential phase is the most active period of toxin production, followed by the stationary phase, with little to no toxins produced in decline phase. The cell-free filtrate displayed relatively low (<10%) and steady inhibition rates in the exponential, stationary and decline phases, indicating that a small amount of toxins is secreted from algal cells during the growth of *A. sanguinea*, which could remain stable in culture medium. There are similar studies showing that cells of *Chattonella marina*, *Karenia mokimotoi*, *Heterosigma akashiwo* and *Prymnesium parvum* exhibit the highest hemolytic activities on rabbit erythrocytes in the exponential phase [17,22], and the inhibition effect of exponential *Pseudo-nitzschia multiseries* culture on *R. salina* is stronger than that in stationary the phase [23].

### 4.3. Action Mode of A. sanguinea Toxicity on Phytoplankton and Zooplankton

Study 3 explored the possible action mode of *A. sanguinea* toxicity to co-occurring phytoplankton and zooplankton by using an osmotic membrane device. The half-contact experiments provided an intuitive way to compare the effects of two action modes (contact and non-contact) on *A. sanguinea* and a target, which mimicked the coexistence of the two modes under natural circumstances and explored the possible ecological significance to *A. sanguinea* populations.

When *R. salina* was used as target, contact and non-contact co-culture experiments showed that *A. sanguinea* JX13 could inhibit the growth of *R. salina* under the two co-cultured modes, while growth promotion in JX13 occurred only in the contact coculture (increased by 50%, Figure 5A). The half-contact experiment showed that *A. sanguinea* JX13 could inhibit *R. salina* under both contact and non-contact conditions, but contact led to a significant increase in inhibition rate by 30% (*p* < 0.01, Figure 6B). Both contact and non-contact conditions in the half-contact experiment promoted the growth of *A. sanguinea* JX13, while the non-contact experiment did not promote *A. sanguinea* JX13 growth. According to the above observations, *A. sanguinea* cells in the insert could benefit from organic substances released via cell contact with *R. salina* outside of the insert. Correspondingly, there was no significant difference in the cell density of *A. sanguinea* AS2 co-cultured with *R. salina* compared with the control (Figure 5C). The results suggest that the inhibitory effect of *A. sanguinea* JX13 on *R. salina* and the growth-promotion effect on its own are both closely related to toxicity. In conclusion, *A. sanguinea* JX13 can inhibit the growth of co-occurring phytoplankton (allelopathic effects) by releasing toxins (or allelochemicals) with or without contact, yet cell contact can greatly enhance the inhibition on phytoplankton and promote its own growth by utilizing organic substances from lysed targets.

When rotifer was used as target, the contact and non-contact co-culture experiments showed that *A. sanguinea* and *B. plicatilis* were not affected by one another under non-contact coculture, but contact coculture led to a reduction in *B. plicatilis* by 30% and a slight inhibition in *A. sanguinea* JX13, which was possibly caused by feeding (Figure 7A,B). Similar results were obtained from the half-contact experiment. Correspondingly, the growth of *A. sanguinea* AS2 was inhibited by 30% under the two co-culture modes (Figure 7C), while *B. plicatilis* was only slightly inhibited (<20%) under the contact condition (or outside the insert) (Figure 7D and Figure 8D). In contrast with *A. sanguinea* JX13, AS2 was significantly inhibited by *B. plicatilis*, even without contact, suggesting that *B. plicatilis* may release growth inhibitors acting on phytoplankton, while it seemed to have no effect on toxic strain *A. sanguinea* JX13. In general, direct contact was the necessary condition for mortality of rotifer. In addition, *A. sanguinea* AS2 only led to a slight decrease in rotifer (20%), while *A. sanguinea* JX13 caused high mortality (50%), indicating that mechanical injury may only be partially responsible for the death of rotifer, and toxins should play a more important role. These results indicate that the toxic *A. sanguinea* strain obviously had a greater competitive advantage than the nontoxic strain.

Many studies have shown that the dinoflagellate *A. sanguinea* has mixotrophic ability [15,16,24,25]. In study 2 and study 3, the growth of toxic strains *A. sanguinea* JX13 and JX14 was significantly promoted under contact with *R. salina*, while the nontoxic strain *A. sanguinea* AS2 was not, which demonstrates that the toxicity of *A. sanguinea* is related to the mixotrophic mode of *A. sanguinea*. Yang et al. (2021) found that *A. sanguinea* strains with higher toxicity also showed stronger mixotrophic capacity [16]. In addition, previous studies have shown that *A. sanguinea* can grow heterotrophically by phagotrophy in the presence of prey or lack of nutrition [24]. *A. sanguinea* can also obtain nutrients by swallowing target algal cells, such as *H. akashiwo*, *R. salina* and *Alexandrium tamarense* [26]. Recent studies have shown that some harmful dinoflagellates can kill aquatic animals by micropredation with the aid of toxins [27,28,29]. For example, Vogelbein et al. (2002) found that fish mortality during *Pfiesteria shumwayae* blooms resulted from micropredatory feeding [30], and *Cochlodinium polykrikoides* [31] and *Karlodinium armiger* [27] caused death of bivalve larvae and copepod by direct contact with live cells. Song et al. (2020) also suggested that micropredation was the key factor responsible for the mass mortality of fish during blooms of *K. australe* [28]. Based on this study, the mixotrophic dinoflagellate *A. sanguinea* may exhibit micropredation behavior, and direct contact is the key factor to trigger the release of toxins.

## 5. Conclusions

Our study demonstrates that *A. sanguinea* JX13 and JX14 present acute toxicity to zooplankton *B. plicatilis*. Toxic effects of *A. sanguinea* JX13 and JX14 on *R. salina* were significantly stronger than those of AS2. The inhibition rates of cell pellet were much higher than those of cell-free filtrate in the exponential and stationary phases, which means *A. sanguinea* cells should be the source of toxins, while a small amount of toxins was secreted from algal cells during growth. Osmotic membrane experiments showed that *A. sanguinea* JX13 displayed an inhibitory effect on coexisting phytoplankton whether they had contact or not but that the lethal effect of *A. sanguinea* JX13 on zooplankton only happened in the contact treatment. Direct contact is the key mode for *A. sanguinea* to release toxins and cause toxic effects on co-occurring plankton.

## Data Availability

Data are available upon reasonable request. Please contact the contributing authors.
